# Land use effects of biofuel production in the US

**DOI:** 10.1088/2515-7620/acd1d7

**Published:** 2023-05-12

**Authors:** Weiwei Wang, Madhu Khanna

**Affiliations:** 1 School of Business, Nanjing University of Information Science and Technology, Nanjing 210044, People’s Republic of China; 2 Department of Agricultural and Consumer Economics, University of Illinois at Urbana-Champaign, Champaign, IL 61801, United States of America

**Keywords:** soybean biodiesel, corn ethanol, corn oil biodiesel, economic optimization model, induced land use change

## Abstract

Biodiesel production from soybean has been growing in the United States and although its amount is small by comparison with corn ethanol, its addition to existing demands on land can have nonlinear effects on land use, due to an upward sloping and increasingly inelastic supply of land. It is critical to quantify these effects to inform future policies that may expand production of soy biodiesel. Here we apply a multi-period, partial equilibrium economic model (BEPAM) to determine land use under a validated counterfactual scenario with no biofuel policy or with corn ethanol mandate alone to isolate the extent to which expansion of biodiesel production in the US led to the conversion of Conservation Reserve Program (CRP) acres and other noncropland to crop production, over the 2007–2018 period. We find that the land use change intensity of biodiesel ranged from 0.78 to 1.5 million acres per billion gallons in 2018 which is substantially higher than that of corn ethanol, that ranged from 0.57 to 0.75; estimates at the lower end of these ranges are obtained under the assumption that there is no conversion of permanent pastureland to cropland and better supported by model validation than the upper end of these ranges. The land use change elasticity with respect to changes in land rent was more inelastic for biodiesel than for corn ethanol. The largest levels of expansion in cropland were in Iowa, Minnesota, North Dakota, Kansas, Michigan and Mississippi.

## 1. Introduction

The growth in biofuel production from food crops, primarily corn and soybeans in the U.S., has been accompanied by concerns about its impact on prices of food and feed and competition for land that can lead to conversion of non-cropland to crop production (Searchinger *et al* 2008, Austin *et al* 2022, Lark *et al* 2022). While much of the biofuel production in the US has been from corn, the production of biodiesel from soy, corn oil and FOGs (fats, oils and grease) has been increasing. Over the same period, studies show that there has been an expansion in cropland acres (USEPA 2018, Lark *et al* 2020). This includes land enrolled in the Conservation Reserve Program (CRP) that was retired from crop production for environmental reasons. USDA’s Farm Service Agency data indicate that total land enrolled in the CRP declined from 36.7 million acres in 2007 to 22.6 million acres in 2018. While the demand for total acres for enrollment in CRP is determined by the annual enrollment cap set by Farm Bill, the supply of land for enrollment in CRP from new and expiring acres will depend on the land rental payments offered by the program relative to the profitability of keeping that land in crop production.

This paper examines the supply-side incentives for expiring CRP acres to re-enroll in the program (assuming they had the option to re-enroll) under various biofuel policy scenarios and the extent to which their exit from the program can be attributed to the biofuel-induced increase in crop prices over the 2007–2018 period. Bigelow *et al* (2020) report that 36% of expiring CRP land during the 2013–16 period was subsequently reenrolled, with 64% choosing to exit the program, but do not identify the factors that led to the exit. While numerous studies have examined the implications of corn ethanol production for land use change (Austin *et al* 2022) and CRP acres (Chen and Khanna 2018) there has been very limited assessment of the effects of additional biodiesel production on land use change in the US. With a convex, upward sloping supply curve for land and a constraint on the amount of arable land available, the elasticity of land use change with respect to land rents is expected to decline as incremental demands for land area increase (Sartori *et al* 2019). Thus, the effects of the addition of biodiesel on land use change, stacked on the demand for land imposed by corn ethanol, can be expected to be different from that of corn ethanol alone. Additionally, differences in soybean and biodiesel yields per unit land relative to corn and corn ethanol are also expected to affect the land use impacts of biodiesel production.

Biodiesel production from soybean and corn oil has grown from 91 million gallons in 2005 to about 1.3 billion gallons in 2018 and it constituted about 7.4% of total biofuel production in 2018. Over this period corn ethanol production grew from 3.9 to 16.1 billion gallons. In examining the extent to which the observed reduction in CRP acres and in other noncropland can be attributed to biodiesel over the 2007–2018 period, it is important to control for the land use change that would have occurred anyway (even in the absence of the increase in biofuel volumes that took place over this period) and for the land use change that can be attributed to corn ethanol alone while accounting for the complex interactions among feed, fuel and co-product markets and the rotation choices with corn and soybeans.

We undertake this analysis by developing a validated counterfactual scenario that controls for the reduction in CRP acres that would have occurred even in the absence of the increase in biofuel production since 2007 and then using this counterfactual to quantify the extent to which the conversion of expiring CRP and other noncropland to crop production can be attributed to corn ethanol and to biodiesel over this period, while keeping all other factors unchanged. In addition to expiring CRP acres, the other noncropland that can potentially convert to crop production includes cropland pasture (defined by the National Agricultural Statistical Service (NASS), as land that is intermittently used for crop production and either in a crop-fallow rotation or used for pasture) and permanent pastureland. We analyze the implications of varying assumptions about the noncropland available for conversion. We apply a multi-period, multi-sector, open economy, partial equilibrium economic model, the Biofuel and Environmental Policy Analysis Model (BEPAM), to conduct a retrospective analysis of the effect of increased biofuel production on the conversion of noncropland to crop production in the US over the 2007–2018 period.

Several studies have examined the effects of using corn for ethanol on land use change using satellite data (Wright and Wimberly 2013, Lark *et al* 2015, Wright *et al* 2017, Lark *et al* 2020), empirical analysis (Miao 2013, Motamed *et al* 2016, Li *et al* 2019, Lark *et al* 2022) and simulation models (Beach and McCarl 2010, Mosnier *et al* 2013, Taheripour *et al* 2017, Khanna *et al* 2020, Taheripour *et al* 2022). Satellite image-based analysis shows a trend of declining grassland acreage (including pasture and hay) and a net increase in cropland from 2008 to 2012 (Lark *et al* 2015) and from 2008 to 2016 (Lark *et al* 2020). Based on land cover data, Wright *et al* (2017) estimate that 2.7 million acres of arable non-cropland were converted to crops within 50 miles of refinery locations between 2008 and 2012. The land use intensity is 0.64 million acres per billion gallons given the 4.2 billion gallons ethanol production expansion during the same period. Li *et al* (2019) examine the extent to which changes in cropland acreage can be causally attributed to changes in corn ethanol production and crop prices. They find that the increase in 4.2 billion gallons of ethanol capacity led to a 2.1 million acre increase in total crop acreage during the 2008–2012 period. The resultant land use intensity is 0.5 million acres per billion gallons. Most recently, Lark *et al* (2022) analyzed the effects of expansion of corn ethanol on crop prices and its consequences for land use change and the environment. They find that 5.5 billion gallons of additional ethanol production led to 5.2 million acres expansion of total cropland over 2008–2016 (0.95 million acres per billion gallons). Their analysis relies on estimating a series of reduced form econometric models and determining the effect of corn ethanol on crop prices separately from the effects on land use change. In contrast, our analysis here estimates the land use effects of biofuels simultaneously with their effects on crop prices and explicitly isolates the effect of corn ethanol and biodiesel on crop prices and land use change while distinguishing between the effects on CRP and other noncropland.

While large-scale general and partial equilibrium numerical models have been used to simulate the effects of biofuel policies on food prices and land use change, these studies have either assumed that land enrolled in CRP is fixed at a given level or have not specifically examined the implications for CRP enrollment (see Beach and McCarl 2010, Khanna and Zilberman 2012, Taheripour *et al* 2017). An exception is Chen and Khanna (2018) that examines the effects of corn ethanol on incentives for CRP conversion. They found that the increase in corn ethanol production from 6.5 to 13.2 billion gallons led to the conversion of 3.2 million acres of unused cropland (0.48 million acres per billion gallons). Most of economic simulation studies estimate the effects of increase in corn ethanol, and some studies explicitly include soybean biodiesel (Cai *et al* 2013, Taheripour *et al* 2017, Taheripour and Tyner 2020). However, these studies only examine the land use change effects due to combined biodiesel and corn ethanol shocks. There have been few of estimates (either simulation or empirical) of land use change effects attributable to biodiesel alone (Austin *et al* 2022). An exception is Chen *et al* (2018) that analyzes the effects of a one-time expansion in soy biodiesel by 0.8 billion gallons and finds that it decreases cropland pasture acreage by about 0.7 million acres (0.9 million acres per billion gallons); they do not control for the production of corn ethanol or provide a comparable estimate of land use impacts of ethanol. This paper extends the analysis in Chen and Khanna (2018) that applied BEPAM to examine the effects of corn ethanol production on conversion of CRP and cropland pasture to crop production over the early period of 2007–2012 in several ways. First, this paper extends BEPAM to include detailed representation of biodiesel production from soybean and Distillers Dried Grain Solubles (DDGS)-based distillers corn oil to examine the incremental effects of biodiesel production on land use change over the 2007–2018 period. Second, we expand the analysis to study the potential for pastureland, as another category of noncropland (in addition to cropland pasture and CRP), to convert to crop production. Third, we extend the analysis of the effects of corn ethanol on land use from the 2007–2012 period to 2007–2018 in order to control for the effects of corn ethanol on land use over this longer period.

In this study, we analyze the extent to which biodiesel production led to an additional increase in crop prices beyond the level that was induced by corn ethanol alone and induced the conversion of various types of land to crop production. Our multi-period optimization model enables us to incorporate the choice for land enrolled in CRP with expiring contracts to return to crop production or re-enroll in the program by comparing the future stream of returns to land between the two choices. The model is validated by comparing simulated CRP land conversion due to biofuel mandates with the observed data on loss of CRP acres over the 2007–2018 period. We then use this validated model to explore land use change with and without biofuel production, holding all other modeling assumptions constant across scenarios. Our analysis contributes to the existing literature by isolating the effects of corn ethanol and biodiesel on the change in land use from CRP and other land to crop production. We estimate the elasticity of land use change due to higher prices induced by biodiesel production and compare that to the elasticity of land use change due to higher prices induced by corn ethanol.

## 2. Methods

### 2.1. Modelling framework

BEPAM is a multi-period, multi-market equilibrium model which determines the production and consumption decisions and the optimal land allocation to various crops by maximizing the discounted sum of consumers’ and producers’ surpluses in the agricultural and transportation sectors subject to various material balance, technological, land availability, and policy constraints over the 2007–2018 period (Chen *et al* 2014, Chen and Khanna 2018). The agricultural sector in the model includes markets for thirteen primary crops, eight types of livestock products, and various processed commodities. Primary crop and livestock commodities in the model can be consumed either domestically or traded with the rest of world (exported or imported). The commodity domestic demand functions and export demand functions for tradable row crops and processed commodities are assumed to be shifted upward over time. The rates of these shifts in demand and the elasticities used for calibrating demand and supply curves are reported in Chen *et al* (2014). The transportation fuel sector includes markets for gasoline, diesel, three types of biofuels (corn ethanol, soy biodiesel and corn oil biodiesel) and their by-products (e.g., DDGS). Soybean oil and distillers corn oil extracted from DDGS are two main biodiesel pathways considered in the model. In the analysis in this study, we focus on the effects on the agricultural sector of given levels of biofuel production and do not consider effects on gasoline and diesel production.

To analyze the land use effects of expanded biofuel production on land use change, we model the effects on use of corn and soybean and the co-products of biofuels for livestock feed. Co-products generated during the production of corn ethanol in the form of DDGS can be used as livestock feed as a replacement for corn and soybean meal. DDGS can be used domestically for feeding livestock and the rest of the DDGSs is exported to the world market.

We include the production of several types of livestock in BEPAM. The model includes demand functions for various finished livestock products: beef, chicken, turkey, lamb, pork, wool, dairy and eggs. A production function is defined for each type of livestock which relates the number of units produced to the quantity of protein and starch consumed. These nutrients are derived from corn, soybeans, alfalfa, and DDGS. To prevent unrealistic changes in production levels, the quantity of various types of livestock produced is constrained by historical maximum numbers at the national level. The historical livestock numbers at the national level are obtained from NASS. Two-year (2006–2007) average prices, domestic consumptions, exports and imports of livestock commodities and assumed elasticities of demand and supply are used to calibrate the domestic and export demand and import supply functions. Livestock production uses alfalfa hay and primary crop commodities like corn, sorghum, soybeans, as feed and byproducts of processing crops such as soybean meal.

The simulation model considers spatial heterogeneity in crop and livestock production across 295 crop reporting districts (CRDs) in 41 US states. The model includes five distinct types of land, namely regular cropland, cropland pasture, land enrolled in CRP, permanent pastureland, and forest pastureland. These and other definitions are in the SI (table S1). We assume that a given type of land in a CRD is homogeneous in its productivity. Cropland can move freely between the production of alternative crops without extra cost. We assume that noncropland can convert to row crops with a conversion cost and that this conversion cost is higher than the returns from crop production in the base year to rationalize this land remaining as noncropland. Corn and Soybeans can be produced using alternative tillage (conservation tillage and conventional tillage), rotation, and irrigation practices. Costs of fertilizer, chemicals and machinery under conventional tillage differ from those under conservation tillage. Production costs and yields of individual crop activities are specified differently for each CRD based on crop budgets data reported by state extension organizations and NASS database.

The BEPAM is a forward-looking model with 10-year rolling horizon based on the assumption that landowners make land allocation plans for the next ten years each year (2007–2016, 2008–2017, and so on). This is particularly true for land enrolled in CRP because CRP contracts typically last for 10–15 years. Specifically, the model first solves for the 2007–2016 period; we then take the first-year solution values such as land allocation among different uses and crop prices as ‘realized’, move the horizon one year forward and solve the new problem, and iterate until the problem is solved for year 2018 (thus, to solve for outcomes in 2018, we conduct the last simulation for the period 2018–2027). The demands for corn ethanol and biodiesel for each year of the 10-year period in each iteration are specified exogenously and fixed in accordance with the specified levels of production of these two biofuels in each of the policy scenarios. Demand curves are fixed within a rolling horizon and updated before the next rolling horizon to incorporate growth in demand over time. Similarly, parameters such as total land availability, noncropland productivity, crop yields, production costs, land conversion costs and conversion efficiency of biofuels are assumed to remain fixed within a rolling horizon. Parameters such as the expiring CRP acres and the biofuel production target are set to change in accordance with the observed values within the 10-year rolling horizon. Some of the parameters such as crop yields, expiring CRP acres, biofuel production target, amount of expiring CRP that converts to cropland acreage, CRP land rental payments, production costs are updated before moving to the next iteration run. A key contribution of our modeling approach is that it incorporates spatial and temporal heterogeneity in agricultural crop yields, costs of production, availability of cropland and noncropland at a CRD level for each of the 295 such districts in the US. More details on BEPAM model can be found in Chen *et al* (2014) and Chen *et al* (2021). Here we apply this model to simulate land-use decisions under the three alternative scenarios described below.

### 2.2. Scenarios simulated

To isolate the impact of soy and corn oil biodiesel production on the expansion of cropland and on crop prices, we simulate three scenarios with the BEPAM that differ in their levels of ethanol and biodiesel production, while keeping all other modeling assumptions the same. We use 2007 as the starting year for our analysis with BEPAM because the model was parameterized with 2007 prices and land use data and 2008 was the first year of a binding biofuel mandate after the establishment of the RFS. We examine the effects of additional biofuel production relative to 2005 levels of production.

- Scenario 1 (No Biofuel Policy Scenario): EtOH and BD 2005. In this scenario, corn ethanol (EtOH) production is maintained at the 2005 level of 3.9 billion gallons and biodiesel (BD) production from soybean oil and DDGS corn oil, at the 2005 level of 91 million gallons for the duration of the 2007–2018 period.
- Scenario 2 (Corn Ethanol Mandate Scenario): EtOH Observed and BD 2005. In this case, corn ethanol production increases from 6.5 to 16.1 billion gallons over the 2007–2018 period as observed while biodiesel production remains at the 2005 level over this period.
- Scenario 3 (Corn Ethanol and Biodiesel Mandate Scenario): EtOH and BD Observed. In this scenario, the combined production of biodiesel from soybean oil and DDGS corn oil increases from 0.4 billion gallons in 2007 to 1.3 billion gallons in 2018 and corn ethanol production increases as in Scenario 2.

We compare outcomes in Scenario 2 with Scenario 1 to estimate the extent to which the increased demand for corn ethanol after 2005 led to an increase in crop prices and land use change during the 2007–2018 period and similarly compare outcomes in Scenario 3 with Scenario 2 to estimate the additional effects of increased demand for biodiesel after 2005. Our analysis incorporates the varying availability of CRP acres with expiring contracts in each year as these acres have the option to re-enroll in the program or to return to crop production. We also analyze the validity of the model with and without the inclusion of permanent pastureland as potentially convertible to crop production and find that its inclusion worsens model fit.

### 2.3. Data

The annual levels of biofuel production are shown in figure S1. Production of corn ethanol increased from about 3.9 billion gallons in 2005 to 16.1 billion gallons in 2018. Production of soybean biodiesel increased from 91 million gallons in 2005 to 1 billion gallons in 2018 while that of distillers corn oil increased from zero to 278 million gallons in 2018. The cost of corn ethanol includes the cost of corn including the costs of inputs and field operations and a cost of land. The former are calculated at county level for each crop using data and methods described in Chen *et al* (2014), while the value of land rents are endogenously determined by the model to equate the demand and supply of land. Technological parameters for converting corn to ethanol and the industrial cost of processing corn to ethanol are described in Chen *et al* (2014). The conversion efficiencies (yield of biofuel per metric ton of feedstock) are exogenously fixed and based on the estimates in GREET 2020 (Argonne National Laboratory 2020); the impact of improvements in this over time are analyzed in the sensitivity analysis. We assume that the share of ethanol produced from dry mill plants is 86% in 2007 and grows over time and that the conversion efficiencies of dry mill plants is 97.4 gallons denatured ethanol per ton of corn, while that of wet mill plants is 93.8 gallons of denatured ethanol per (US) ton of corn (Mueller 2010). The cost of conversion of corn grain to ethanol is estimated as $0.76 per gallon in 2007 prices net of co-product credits (EPA 2010).

The production level of biodiesel from soybean oil and DDGS-based distillers corn oil is specified exogenously in accordance with the observed production level for the 2007–2018 period. Feedstock costs for soybean biodiesel are assumed to be endogenously determined at the market prices of soybean oil. The conversion rate of soybean oil to biodiesel is assumed to be 259.4 gallons per (US) ton of soybean oil (Antares Group Inc. 2009). Distillers corn oil extracted from DDGS is added as a biodiesel pathway as part of the dry milling process. Corn oil from extraction is nonfood grade and can only be used for biodiesel production in the model. Wet mill corn oil and corn oil fractions are not considered in the model, given that neither of them is an approved pathway for compliance with the RFS. The conversion coefficient of DDGS to corn oil is assumed to be 19.5 gallons of corn oil per (US) ton of DDGS. The cost of extracting oil from DDGS is $0.05 per gallon of corn oil produced and the processing cost of converting oil from DDGS into biodiesel is assumed to be $0.26 per gallon of corn oil. The cost of DDGS extracted oil is assumed to be $1.25 per gallon (Beach and McCarl 2010). 92% of reduced-oil DDGS can be reused as livestock feed with 2% less feed value than full-oil DDGs (Office of the Federal Register 2018). The price of DDGS is determined by the lagged prices of corn and soymeal as described in Chen *et al* (2014).

Land availability data for different land types for each county were obtained from the USDA/NASS and further aggregated to the CRD level. Planted acres for the thirteen conventional crops was 304 million acres in base year 2007; this accounts for 91% of total cropland (under all crops (row crops, hay, silage, fruits and specialty crops) and categories (harvested, idle, fallow and failed)) in the US in 2007. It accounts for 96% of the cropland area to the east of the Great Plains region where most crop-based biofuels are produced. Observed availability of cropland pasture was 37.6 million acres in 2007, while the observed availability of pastureland and forestland pasture was 383 and 26 million acres in 2007, respectively. County-level CRP contract data was obtained from the Farm Service Agency of the US Department of Agriculture. The dataset included CRP contracts for 3069 counties in lower 48 states from 1997 to 2018 and the average rental rate at each signup as well as the total CRP acres enrolled in each county (continuous and general enrollment). Observed total CRP enrollment was 36.7 million acres in 2007. The average rental rate is the area-weighted average of rental payments for CRP land parcels in continuous and general enrollment. The county-level CRP data was aggregated to the CRD level for ease of numerical simulation. In the case of expiring CRP acres, the opportunity cost of its conversion to cropland is the discounted value of the sum of the land rental payments they can receive from re-enrollment over a 10-year period. These land rental payment for each CRP contract are closely related to the county-specific dryland rental rates, with adjustments for relative soil productivity (Stubbs 2014). If the net returns from the conversion of these acres to crop production are larger than the discounted value of the soil rental payments from reenrolling in CRP, then the CRP acres are assumed to convert to crop production. This approach assumes farmers are risk neutral and do not value the certainty of CRP payments relative to the risky returns from converting the expiring acres to crop production; we may therefore be overestimating incentives for conversion of expiring CRP acres to cropland if farmers are typically risk-averse.

In the absence of publicly available information on the productivity of CRP acres and their exact location, we approximate the ratio of the productivity of CRP to regular cropland acres by the ratio of the rental payment for a CRP acre to the average dryland cash rents in each CRD (as in Chen and Khanna 2018). The net returns from converting expiring CRP acres and other noncropland to cropland are endogenously determined in the model and increase as crop prices increase with heightened demand for crops as biofuel feedstocks. This estimated crop productivity ratio varies by CRD and averages 36.8% across CRDs (SI figure S2).

We estimated the costs of production in 2007 prices for the thirteen row crops at the county level, which are then aggregated to the CRD level for computational ease. Production costs and yields of individual crop and resource endowments were obtained from various agricultural experiment stations and the USDA/NASS database. We used the historical five-year average (2003–2007) yield per acre for each CRD to calculate average yields of conventional crops for that CRD. The yields of major crops, including corn, soybeans, and wheat, were assumed to increase over time at the exogenous rate estimated using historical data. These yields are also assumed to be price-elastic with the price elasticities estimated econometrically.

## 3. Results

We validate the model by comparing various model simulated outcomes, including CRP acres, total cropland acreage, crop-specific acreages for corn, soybeans, and wheat, crop and fuel prices, production and consumption over time with observed data (Figure S3–S6 in SI). A more detailed description of validation results can be found in the model validation section in SI.

By testing the ability of the model to provide outcomes close to those observed in reality and then keeping all assumptions the same in the counterfactual scenarios and focusing on the deviations in outcomes between the two scenarios, we reduce the effects of uncertainty about these assumptions that affect both scenarios equally on the estimate of this deviation as much as possible. We use this validated model to develop the counterfactual scenarios with no increase in biofuels or with corn ethanol production alone and isolate the land use change effects attributed to increased ethanol and to biodiesel production during the 2007–2018 period. The sensitivity of the model to several assumptions is estimated and reported in the section of sensitivity analysis.

### 3.1. Changes in cropland due to biofuel production

We now present annual results of land use change under the three simulated scenarios (table 1 with no permanent pastureland conversion; corresponding SI table S2 has the results with permanent pastureland conversion). The reduction in expiring acres annually is aggregated over time to estimate the total amount of CRP acres converted to crop production; this land once converted to crop production is assumed to remain under crop). In the case of other noncropland (cropland pasture and permanent pastureland), we assume that it can be intermittently in crop and noncrop; hence we examine the annual decision by this land to be in crop or noncrop use.

In Scenario 1 (EtOH and BD 2005), the simulated annual reduction in CRP acres since 2008 ranged from 0.26 (in 2016) to 1.38 (in 2012) million acres, totaling 9.2 million acres by 2018. Additionally, each year cropland pasture was converted to crop production with an amount that varied annually, ranging from 8.9 million acres (in 2018) to 10.9 million acres (in 2008). As a result, we find that 18.1 million acres of additional land transitions to crop production during the 2007–2018 period even in the absence of the additional demands due to biofuel. In Scenario 2 (EtOH Observed and BD 2005), we find that total amount of noncropland converted to cropland by 2018 amounts to 24.9 million acres. This implies that 6.9 million acres of additional noncropland converted to crop production due to the corn ethanol production from 2007 to 2018. In Scenario 3 (EtOH and BD Observed), with the addition of the soybean and corn oil biodiesel to Scenario 2, the total amount of CRP acres that exit the program is 13.6 million acres. This together with the 12.3 million acres of cropland pasture that convert to crop production amounts to 25.9 million acres of noncropland converting to crop production in 2018. This implies that an additional 0.92 million acres of noncropland converted to cropland in 2018, of which 0.78 million acres were expiring CRP acres and 0.14 million acres were cropland pasture. These estimates indicate that 38.3% of the total noncropland and 40.0% of CRP acres that converted to cropland in Scenario 2 in 2018 can be attributed to corn ethanol production. In 2018, another 5.1% of the noncropland and 8.5% of the CRP acres converted to crop production can be attributed to the additional production of biodiesel from soybean and corn oil. In the case with pastureland conversion, the total amount of noncropland conversion is much higher (1.6 million acres) but the amount of CRP acres converted was much smaller (0.67 million acres) while the amount of other noncropland conversion was much larger (0.95 million acres) relative to the land use change attributed to biodiesel without pastureland. The overall estimates indicate that 48.6% of the total noncropland and 36.4% of CRP acres that converted to cropland in 2018 can be attributed to corn ethanol production. Biodiesel production leads to another 8.7% of noncropland and 8.8% of CRP acres conversion (table S2).

**Figure 1.**
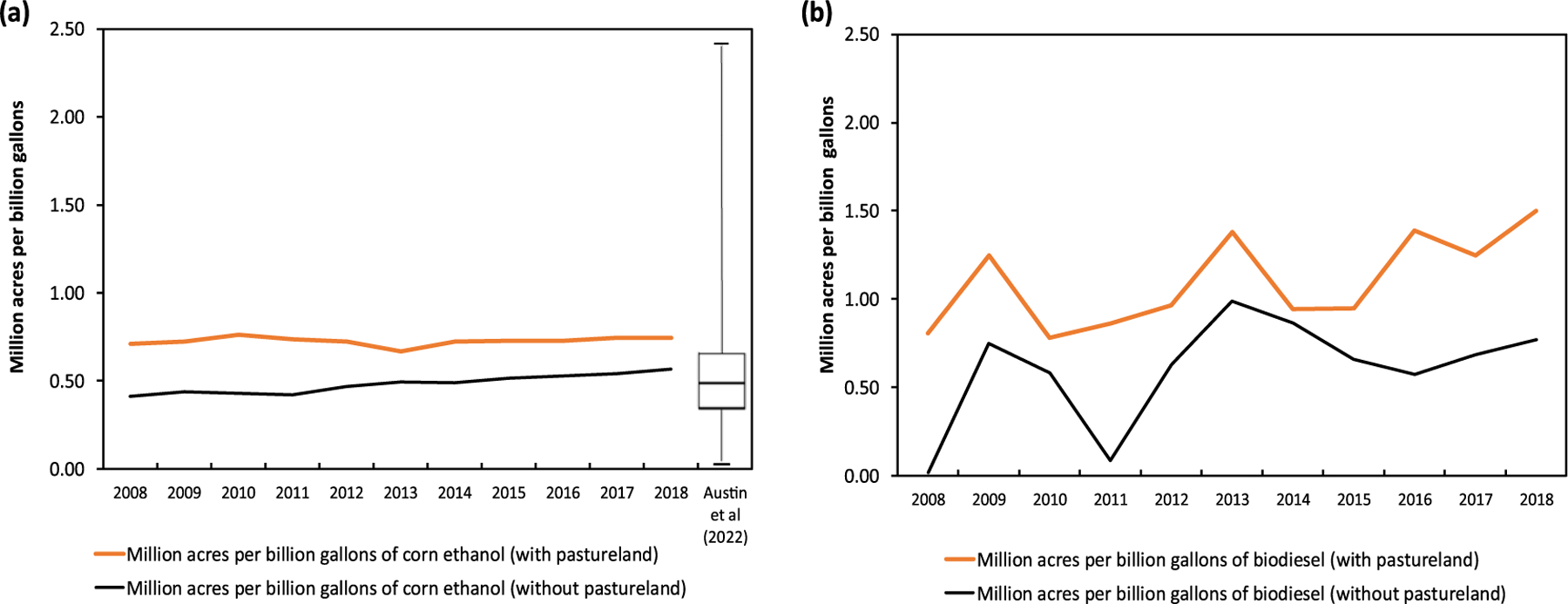
Expansion in cropland per unit corn ethanol and per unit biodiesel. (a) land expansion due to corn ethanol production; (b) land expansion due to biodiesel production. Black lines represent benchmark scenario with no pastureland. Orange lines represents alternative scenario with pastureland. The box-whisker plot in figure 1(a) denotes the range of million acres per billion gallons of biofuel estimated by existing literature as summarized in the review paper by Austin *et al* (2022). The minimum and maximum value is 0.01 and 2.47, respectively. The median value is 0.47.

We estimated the impact of a biofuel production shock by calculating the change in total cropland per unit of the annual increase in corn ethanol production in each year (2008–2018). As shown in figure 1, this ranged between 0.41 and 0.57 million acres of cropland conversion per billion gallons (as shown in black line in figure 1(a)) without inclusion of pastureland; the corresponding estimate with inclusion of pastureland is 0.71 to 0.75 million acres per billion gallons. The estimate (without pastureland) is close to the estimates ranging between 0.45 and 0.4 million acres per billion gallons obtained in Chen and Khanna (2018). A review of the literature by Austin *et al* (2022) found a median estimate of 0.47 million acres per billion gallons with the recent estimate by Lark *et al* (2022) of 0.94 million acres per billion gallons being at the upper end^3^. Our estimate of simulated land use change intensity (without pastureland) is also in close agreement with the empirically-based estimates of land use intensity in Li *et al* (2019) of 0.43 million acres per billion gallons (2008–2014).

Unlike corn ethanol, the additional annual land requirement per unit biodiesel varies considerably from year to year. This could be due to the year-to-year fluctuations in biodiesel production which were several times larger than those with corn ethanol, lower yields of soybean (relative to corn yields) and the non-linearities in land use change. Biodiesel production requires 0.78 million acres per billion gallons of biodiesel without pastureland (corresponding estimate with pastureland is 1.5 million acres per billion gallons of biodiesel, as shown in figure 1(b)). We estimate that the quadrupling of corn ethanol production led to an increase in total cropland used for crop production by 2.4% in 2018 relative to the counter-factual level in 2018 under Scenario 1 (table 2).

Despite the increase in total corn acreage, there was a net reduction in corn acres to meet food/feed needs relative to 2007. There was also a reduction in acres under soybeans by 11.2% and in land under other food and feed crops by 3.5%. The addition of demands imposed by biodiesel production increases total cropland by 0.9 million acres (0.3% of total cropland); of this 85.4% is met by bringing noncropland into crop production and the rest by keeping cropland (in 2007) in crop production that would have otherwise become idle. Land under soybeans increased by 6.9% relative to Scenario 2 (EtOH Observed and BD 2005). This was partly met by reducing demand under corn and other crops and partly by reducing the use of soybeans for food/feed by 14.5%.

Demand for corn ethanol by 2018 increased land rent by 29.8% compared to Scenario 1 (EtOH and BD 2005). The addition of the demand for biodiesel in Scenario 3 (EtOH and BD Observed) led to a further increase in land rent by 6.6% compared to Scenario 2 (EtOH Observed and BD 2005). Relative to Scenario 1 (EtOH and BD 2005), the increase in demand for corn for ethanol raised corn prices by 31.4% and soybean prices by 20.6% in 2018. The addition of biodiesel production in Scenario 3 (EtOH and BD Observed), further raised corn and soybean prices by 4.3% and 8.2% in relative to Scenario 2 (EtOH Observed and BD 2005), respectively. The implied land use change elasticity of change in total cropland in response to the change in land rents is 7.9% in Scenario 2 (EtOH Observed and BD 2005). This elasticity is 5.1% in Scenario 3 (EtOH and BD Observed); indicating greater inelasticity of total cropland to land rents in response to the increased demand for soybeans compared to that due to corn ethanol. Inclusion of pastureland results in relatively larger land use change elasticity than the case without pastureland. Corresponding values are 11.9% and 5.3% in Scenario 2 (EtOH Observed and BD 2005) and Scenario 3(EtOH and BD Observed), respectively (table S3).

Increased commodity prices induced by corn ethanol production has an important economic implication in that it drives up the land rental payments that need to be offered to prevent expiring acres from exiting the program. We estimate the net present value of rental payments that will need to be paid over a 10-year contract term to prevent expiring CRP acres from exiting the program. Under Scenario 1 (EtOH and BD 2005), the net present value of land rental payments needed to prevent expiring CRP acres from leaving the program for crop production is $23.9 billion. The production of corn ethanol increased CRP maintenance costs by 47% to $35.2 billion relative to the counter-factual Scenario 1. The corresponding maintenance cost in the case with pastureland is about 4.5% less ($33.6 billion) (table S3). The addition of biodiesel targets increases land rents and raises the cost of maintaining CRP acres at the 2007 level by another 5.2% to $37 billion. The corresponding value in the case with pastureland is $35.4 billion.

### 3.2. Regional distribution of land use changes under primary crops

We determine the regional distribution of land use changes under corn, soybeans, wheat and alfalfa in 2018 that could be attributed to the additional corn ethanol production by comparing outcomes under Scenario 2 with those under Scenario 1 (as shown in figures S7 in SI). Most of the increase in corn acres (about 65%) occurred in the states of Iowa, Illinois, Indiana, Minnesota, and Kansas. Cropland under soybeans and wheat was lower by 9.7 and 0.5 million acres in the Midwest, respectively. Corn ethanol production also led to a conversion of 6.1 million acres of noncropland to alfalfa in the Midwest and Great Plains areas. The states included in each region are listed in table S4. We find that the net expansion in corn acreage was 22.2 million acres largely through the substitution of land from other crops (such as soybeans, wheat, alfalfa, etc.) on land that was already under other crops in 2007.

The addition of biodiesel targets to corn ethanol results in some changes in the spatial pattern of crop specific land use change (figure S8). The key changes are a reduction of 0.65 million acres in cropland converted to corn acres in the Midwest and a 2.7 million acres increase in cropland under soybeans in the Midwest. There was also a 2.3 million acres increase in cropland under soybeans in the Great Plains which was achieved largely by reducing cropland under wheat, alfalfa and corn in the same region. We also find that 0.4 and 0.2 million acres of noncropland is converted to alfalfa in the Midwest and Great Plains, respectively, compared to Scenario 2 (EtOH Observed and BD 2005). Overall, we find that the biodiesel production led to a net increase of 4.9 million acres of soybeans, which was largely met by converting cropland under corn, wheat and alfalfa in the Midwest and Great Plains.

### 3.3. Spatial distribution of land use changes

**Figure 2.**
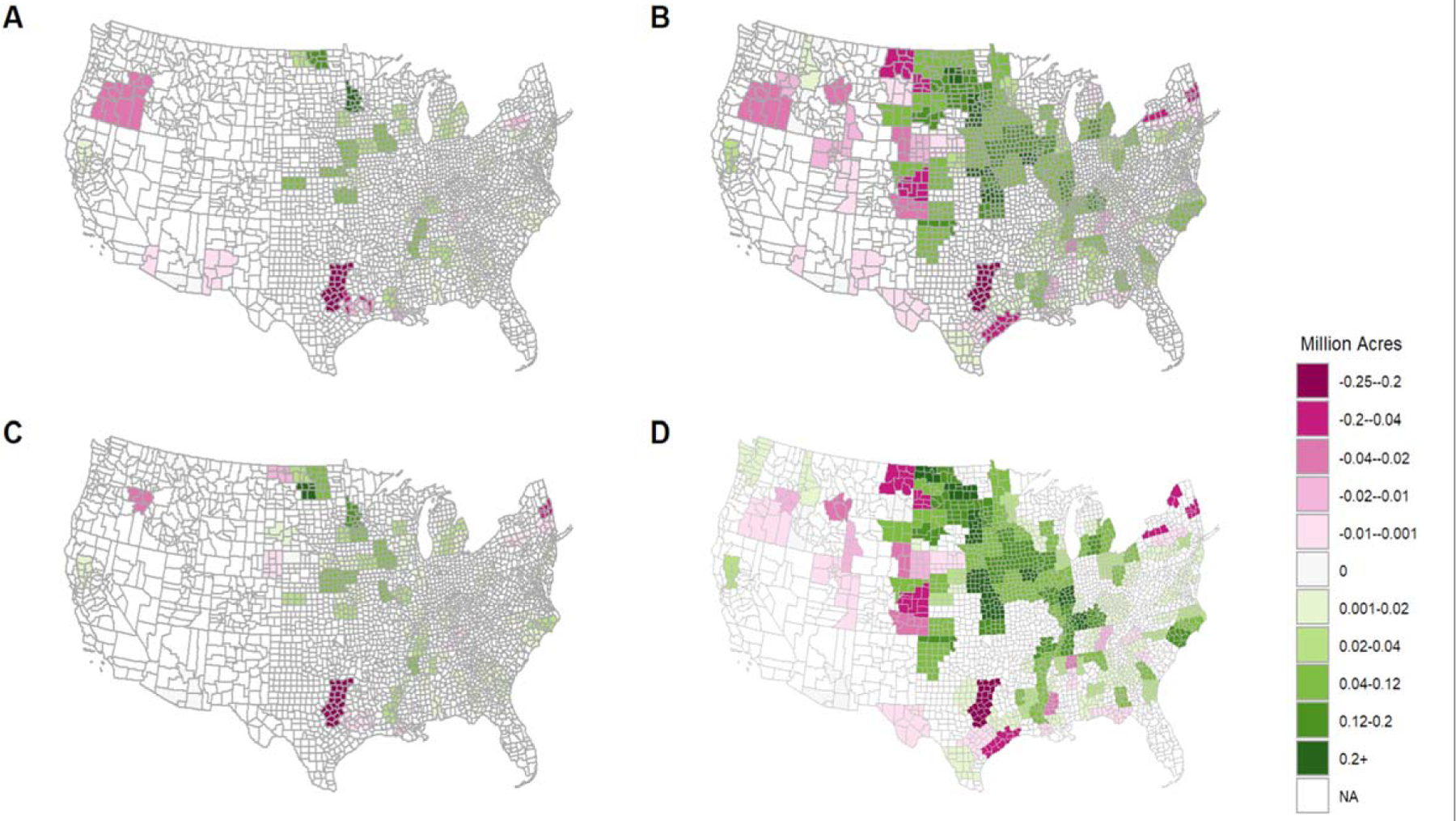
Conversion of noncropland to crop production in 2018. (A) Conversion of noncropland due to biodiesel alone in scenario without pastureland. (B) Conversion of noncropland due to both biodiesel and corn ethanol production in scenario without pastureland. (C) Conversion of noncropland due to biodiesel alone in scenario with pastureland. (D) Conversion of noncropland due to both biodiesel and corn ethanol production in scenario with pastureland. (Note: We display the CRD-level results in the county map by showing the same value across all counties in the same CRD ).

We find that the spatial distribution of the converted noncropland was concentrated in regions with comparative advantage in producing corn and soybeans. The biodiesel production led to much smaller changes in land use and these were more dispersed across the Midwest and the south and south-east. The largest levels of conversion of noncropland to cropland were in Iowa, Minnesota, North Dakota, Kansas, Michigan, Mississippi, Alabama, and Kentucky (figure 2(A)). The additional corn ethanol production after 2005 led to conversion of noncropland to cropland in the Midwest and Northern Great Plains region. Specifically, changes in land use were largest in Iowa, Illinois, North and South Dakota, Kansas, Minnesota, Kentucky, and Nebraska (figure 2(B)). Inclusion of pastureland conversion leads to larger amount of noncropland conversion relative to the case without pastureland. Increased conversion of noncropland is found to occur mainly in the Midwest and Great Plains (in Illinois, Iowa, North Dakota, Kansas and Kentucky) (figures 2(C) and (D)).

### 3.4. Sensitivity analysis

We examined the sensitivity of our key results to wide variation in assumed values of parameters in thirteen cases (table S5 has detailed description of each case and figure S9 has the results). We find that halving the assumed growth rates of yields of corn and soybeans would result in 0.7% higher increase in corn prices, 0.5% higher CRP maintenance costs increase land use intensity of corn ethanol by 4.2%. A ±10% increase in the rental payment for enrolling in CRP would lead to ±6% and ±2% variation in the CRP maintenance costs that can be attributed to corn ethanol and biodiesel production, respectively. The effects of CRP rental payments on other results such as land use change intensity and crop prices are moderate, ranging between −3% and 2%. Land use intensity of biodiesel could be about 9% higher or 9% lower with a halving of the growth rate of yields or a 10% increase in cost of conversion of cropland pasture. Overall, the variation in key results is within ±10% of the benchmark values despite wide variations in various parametric assumptions considered in the sensitivity analysis. Detailed description and findings of sensitivity analysis are in the SI.

## 4. Conclusions and policy implications

This paper estimated the extent to which the expansion of cropland due to conversion of noncropland from the CRP, cropland pasture and permanent pastureland could be attributed to the increase in corn ethanol and biodiesel production over the 2007–2018 period in the US. We determine the increase in corn ethanol by 12.2 billion gallons and biodiesel by 1.2 billion gallons by 2018 (compared to levels in 2005) led to an expansion of 7.8 and 10.9 million acres of cropland by 2018, without and with the inclusion of pastureland acres, respectively. Model validation suggests the scenario without the availability of permanent pasture is more realistic. Excluding permanent pasture, of the increase in cropland by 7.8 million acres, 4.4 million acres were from expiring CRP acres and the rest from other noncropland. We further isolate the extent to which this increase was due to corn ethanol and from biodiesel and find that 3.7 million acres of CRP acres could be attributed to the increase in corn ethanol and 0.7 million acres to the increase in biodiesel, in the case without pastureland conversion. The inclusion of potential conversion by pastureland lowers the extent of conversion of expiring CRP acres and increases the conversion of other noncropland. Overall, we find that just under half (48% without and 45% with pastureland conversion) of the conversion of expiring CRP acres by 2018 can be attributed to biofuels.

The amount of cropland expansion that can be attributed to the production of 1.2 billion gallons of biodiesel is 0.92 million acres without pastureland conversion and 1.62 million acres with pastureland conversion in 2018. We also find that the amount of land conversion per unit biofuel varies non-linearly over time, particularly for biodiesel and ranges from 0.65 to 0.78 million acres per billion gallons in the case without pastureland and 0.95 to 1.5 million acres per billion gallons with pastureland conversion between 2015 and 2018, as additional biodiesel production increase from 0.8 to 1.2 billion gallons (relative to 2005 levels). The range of estimates for corn ethanol are 0.41 to 0.57 and 0.71 to 0.75 million acres per billion gallons without and with pastureland conversion as additional corn ethanol production (relative to 2005 level) increased by 0.5 billion gallons to 12.2 billion gallons between 2008 and 2018. The estimates for corn ethanol without pastureland conversion are close to the median value in the literature reported in Austin *et al* (2022) and very similar to the estimates obtained empirically in Li *et al* (2019), while those with pastureland conversion are within the 75th percentile of estimates in the literature.

We find a low responsiveness of aggregate crop acreage to changes in biofuel-induced crop price increases and a relatively low elasticity of land use change to land rents of 7% with both the corn ethanol and biodiesel mandates. Land use change was somewhat more elastic to the corn ethanol mandate (7.9%) than to the biodiesel mandate (5.1%). Our findings indicate that much of the increased demand for corn and soybeans due to the mandates was met by substitution of land from other crops to corn and soybeans than by conversion of noncropland.

The modeling framework applied here simulates changes in cropland acres over time depending on endogenously determined land returns and food/feed prices, historical allocation of cropland, and projections of demand for food. This study is different from the econometric model-based studies that consider a one-time change in cropland instead of the temporal trend in land use change (Lark *et al* 2022). This approach also differs from that used in existing partial and general equilibrium models that assume a constant elasticity of land supply or land conversion that does not vary over space or time (Hertel *et al *2010, Khanna and Crago 2012).

Our analysis has several implications for policy. First, it is essential for policymakers to consider the full scope of benefits and costs of policy when making policy choices. The biofuel mandates increased food crop prices and land rents, which led to less incentives for enrollment/re-enrollment in CRP at existing rental rates. This implied that higher rental payments for CRP would need to be offered in order to maintain CRP acreage. These additional amounts are not insignificant (e.g., an $11 billion increase in the discounted value of the CRP payments would be needed to maintain CRP and an $1.8 billion increase in the corresponding value due to biodiesel production). Second, we show that on a per unit basis, the indirect effects of corn ethanol and biodiesel production on land use are increasing over time as biofuel production is increasing despite the increase in crop yields over time. Additionally, we show that biodiesel is two times more land intensive than corn ethanol. This implies that expanding biodiesel production to levels close to corn ethanol may have significantly larger adverse consequences for land use, crop prices and incentives for land to exit CRP. Even though additional biodiesel production was 1.2 billion gallons and only 9.8% of the 11 billion gallons of additional corn ethanol production in 2018 (relative to 2005 level), it accounted for 12%–15% of noncropland that was converted to crop production in 2018. Future policies to promote biofuel need to weigh the GHG mitigation benefits of biofuels with other unintended consequences, including the impact on food crop prices, farmer income, CRP enrollment and program cost and higher food crop prices.

Our analysis relies on several simplifying assumptions such as productivity of noncropland, the response of farming practices to crop prices changes, and the rental payments offered to CRP acres. We also lacked observed data on annual changes in cropland pasture. Nevertheless, our modeling approach enables us to validate the model by comparing observed data on changes in CRP acres and total cropland with model-simulated outcomes and to show these are relatively close. By using this validated model to generate a counterfactual scenario of land use change with the 2005 level of corn ethanol and biodiesel we have confidence in estimates of the extent to which land use changes could be attributed to each of the two types of biofuel production. The close agreement we obtain in our finding of the land use effects of corn ethanol with that obtained from previous empirical studies also adds to our confidence in using this framework to assess the land use effects of biodiesel. The model can be extended to predict land use changes effects due to future biofuel production. We leave that for future work.

## Supplementary Material

Supplement1

## Figures and Tables

**Table 1.** Effect of corn ethanol and biodiesel production on noncropland conversion (Million Acres).

	Scenario 1 (EtOH and BD 2005)		Scenario 2 (EtOH Observed and BD2005)		Scenario 3 (EtOH and BD Observed)		Effect of corn ethanol production	Effect of biodiesel production
	Ethanol at 4 BG and biodiesel at 91 MG		Ethanol at observed levels and biodiesel at 91 MG		Ethanol and biodiesel at observed levels			
	Conversion of CRP to cropland	Conversion of crop- land pasture to cropland	Conversion of CRP to cropland	Conversion of crop- land pasture to cropland	Conversion of CRP to cropland	Conversion of crop- land pasture to cropland	(Scenario 2-Scenario 1)	(Scenario 3-Scenario 2)
2008	0.64	10.90	0.70	13.06	0.70	13.06	2.22	0.00
2009	1.34	10.00	1.50	12.87	1.50	13.04	3.02	0.17
2010	1.29	9.67	1.65	13.12	1.65	13.16	3.82	0.04
2011	1.37	9.30	1.60	12.72	1.55	12.81	3.65	0.03
2012	1.38	9.33	1.98	12.28	2.09	12.56	3.55	0.38
2013	0.77	9.54	1.32	12.19	1.69	12.54	3.21	0.71
2014	0.62	9.55	1.01	12.29	1.01	12.48	3.12	0.20
2015	0.48	9.51	0.82	12.43	0.89	12.49	3.27	0.12
2016	0.26	9.29	0.55	12.37	0.61	12.37	3.38	0.06
2017	0.69	9.11	1.09	12.23	1.22	12.24	3.51	0.14
2018	0.31	8.89	0.59	12.14	0.68	12.28	3.52	0.24
Cumulative reduction in CRP (2008–2018)	9.16		12.82		13.59		3.66	0.78
Total conversion of noncrop-land in 2018 relative to 2007		18.05		24.96		25.87	6.91	0.92
% of Noncropland converted relative to Scenario 1							38.3%	5.1%
% of CRP converted relative to Scenario 1							40.0%	8.5%

Note: Changes in cropland pasture acres shown here are relative to the level in 2007 which are not additive over time; conversion of CRP acres is the annual change relative to the previous year and could be cumulated over time.

**Table 2.** Effects of biofuel production on land use and land rent in 2018.

	Scenario 1 (EtOH and BD2005)	Scenario 2 (EtOH observed and BD 2005)		Scenario 3 (EtOH and BD observed)	
			% Change^a^		% Change^b^
Total Cropland in 2018 (Million acres)	320.5	328.1	2.4%	329.2	0.3%
*Cropland in 2007 remaining in crop production in 2018*	301.1	301.7	0.2%	301.9	0.1%
*Conversion of cropland pasture by 2018*	8.9	12.1	36.6%	12.3	1.2%
*Conversion of CRP land by 2018*	9.2	12.8	39.9%	13.6	6.1%
Land under corn (Million acres)	76.4	98.6	29.1%	97.9	−0.7%
*Corn for food*	68.2	64.3	−5.6%	63.9	−0.7%
*Corn for ethanol*	8.2	34.3	317.4%	34.0	−0.7%
Land under soybeans	80.2	71.2	−11.2%	76.1	6.9%
*Soybeans for biodiesel*	1.4	1.5		16.5	
*Soybeans for food*/*feed*	78.8	69.7	−11.5%	59.6	−14.5%
Other food/feed crops	164.0	158.3	−3.5%	155.1	−2.0%
Corn price ($/bu)	3.0	4.0	31.4%	4.2	4.3%
Soybeans price ($/bu)	7.9	9.5	20.6%	10.3	8.2%
Land rent ($/ha)	315.5	409.5	29.8%	436.5	6.6%
Land use change elasticity		7.9%		5.1%	
Total CRP maintenance costs ($ billion)	23.9	35.2	46.9%	37.0	5.2%

Notes

a This column shows the percentage changes in Scenario 2 relative to Scenario 1.

b This column shows the percentage changes in Scenario 3 relative to Scenario 2.

## Data Availability

The data cannot be made publicly available upon publication because they are not available in a format that is sufficiently accessible or reusable by other researchers. The data that support the findings of this study are available upon reasonable request from the authors.

## Footnotes

3 This differs from the estimate reported in Austin et al (2022) which incorrectly reports their land use change estimate as 2.1 million acres instead of 2.1 million hectares.
