# Supplementary material for: Land use effects of biofuel production in the US
Source: Environ Res Commun. Author manuscript; Available in PMC 2025 Mar 26. (PMC11938799; doi:10.1088/2515-7620/acd1d7)
Supplement: Supplement1 [file NIHMS2010770-supplement-Supplement1.docx]

**Supplementary Information**

**for**

**Land Use Effects of Biofuel Production in the US**

1. **Tables and Figures**

**Table S1. Definition of land types in the model (source: USDA/NASS 2014)**

| Land Type | Definition |
| --- | --- |
| Regular cropland | This category includes cropland harvested, cropland on which all crops failed or were abandoned, cropland in cultivated summer fallow, and cropland idle or used for cover crops or soil improvement but not harvested and not pastured or grazed |
| Cropland pasture | This category includes land used only for pasture or grazing that could have been used for crops without additional improvements. Also included are acres of crops hogged or grazed but not harvested before grazing |
| Permanent pastureland | This category includes grazable land that does not qualify as woodland pasture or cropland pasture. It may be irrigated or dry land. In some areas, it can be a high-quality pasture that could not be cropped without improvements |
| Forest pastureland | This category mainly includes forest, brush-grown pasture, arid woodlands, and other areas within forested areas that have grass or other forage growth |

**Table S2: Effect of Corn Ethanol and Biodiesel Production on Noncropland Conversion Under Scenario with Pastureland (Million Acres)**

|  | Scenario 1 (EtOH&BD 2005)  Ethanol at 4 BG and biodiesel at 91 MG | | Scenario 2 (EtOH Observed&BD 2005)  Ethanol at observed levels and biodiesel at 91 MG | | Scenario 3 (EtOH&BD Observed)  Ethanol and biodiesel at observed levels | | Effect of corn ethanol production | Effect of biodiesel production |
| --- | --- | --- | --- | --- | --- | --- | --- | --- |
|  | Conversion of CRP to cropland | Conversion of cropland pasture to cropland | Conversion of CRP to cropland | Conversion of cropland pasture to cropland | Conversion of CRP to cropland | Conversion of cropland pasture to cropland | (Scenario 2-Scenario 1) | (Scenario 3-Scenario 2) |
| 2008 | 0.53 | 13.48 | 0.58 | 17.27 | 0.59 | 17.43 | 3.85 | 0.17 |
| 2009 | 0.91 | 12.30 | 1.02 | 17.21 | 1.03 | 17.48 | 5.02 | 0.28 |
| 2010 | 1.08 | 11.61 | 1.22 | 18.44 | 1.22 | 18.49 | 6.97 | 0.05 |
| 2011 | 1.04 | 11.29 | 1.14 | 18.24 | 1.14 | 18.64 | 7.06 | 0.41 |
| 2012 | 1.21 | 11.22 | 1.50 | 17.27 | 1.54 | 17.73 | 6.35 | 0.49 |
| 2013 | 0.69 | 11.72 | 1.13 | 16.84 | 1.3 | 17.68 | 5.56 | 1.01 |
| 2014 | 0.63 | 11.78 | 0.94 | 17.84 | 0.95 | 18.28 | 6.38 | 0.45 |
| 2015 | 0.46 | 11.62 | 0.79 | 17.74 | 0.99 | 18.23 | 6.46 | 0.53 |
| 2016 | 0.27 | 11.40 | 0.54 | 17.71 | 0.55 | 18.60 | 6.58 | 0.90 |
| 2017 | 0.52 | 11.15 | 1.07 | 17.49 | 1.13 | 18.23 | 6.88 | 0.8 |
| 2018 | 0.32 | 11.03 | 0.5 | 17.32 | 0.65 | 18.43 | 6.47 | 1.27 |
| Cumulative reduction in CRP (2008-2018) | 7.65 |  | 10.43 |  | 11.10 |  | 2.78 | 0.67 |
| Total conversion of noncropland in 2018 relative to 2007 | 18.68 | | 27.75 | | 29.53 | | 9.07 | 1.62 |
| % of Noncropland converted relative to Scenario 1 |  | | | | | | 48.6% | 8.7% |
| % of CRP converted relative to Scenario 1 |  |  |  |  |  |  | 36.4% | 8.8% |

Note that the changes in cropland pasture acres shown here are relative to the level in 2007; this is unlike the conversion of CRP acres which were the annual changes relative to the previous year and could be cumulated over time. Annual changes in cropland pasture are therefore not additive over time.

**Table S3: Effects of Biofuel Production on Land Use and Land Rent in 2018 Under Scenario With Pastureland**

|  | **Scenario 1**  **(EtOH&BD2005)** | **Scenario 2**  **(EtOH Observed&BD 2005)** | | | **Scenario 3 (EtOH&BD Observed)** | |  |
| --- | --- | --- | --- | --- | --- | --- | --- |
|  |  | | % Change^a^ |  | | % Change^b^ | |
| Total Cropland in 2018 (Million acres) | 320.3 | 330.6 | 3.2% | 332.7 | | 0.6% | |
| *Cropland in 2007 remaining in crop production in 2018* | 300.5 | 301.6 | 0.4% | 301.9 | | 0.1% | |
| *Conversion of cropland pasture by 2018* | 11.0 | 17.3 | 57% | 18.4 | | 6.4% | |
| *Conversion of CRP land by 2018* | 7.7 | 10.4 | 32.5% | 11.1 | | 6.4% | |
| Land under corn (Million acres) | 76.3 | 98.3 | 28.9% | 98.7 | | 0.4% | |
| *Corn for food* | 68.1 | 64.2 | -5.7% | 64.4 | | 0.3% | |
| *Corn for ethanol* | 8.2 | 34.1 | 316.4% | 34.3 | | 0.5% | |
| Land under soybeans | 79.9 | 72.3 | -9.4% | 76.5 | | 5.8% | |
| *Soybeans for biodiesel* | 1.4 | 1.5 |  | 16.5 | |  | |
| *Soybeans for food/feed* | 78.4 | 70.8 | -9.7% | 60.0 | | -15.3% | |
| Other food/feed crops | 164.2 | 160.0 | -2.6% | 157.5 | | -1.5% | |
| Corn price ($/bu) | 2.9 | 3.9 | 33.5% | 4.2 | | 7.4% | |
| Soybeans price ($/bu) | 7.9 | 9.3 | 18.2% | 10.3 | | 10.2% | |
| Land rent ($/ha) | 313.3 | 397.6 | 26.9% | 430.7 | | 8.3% | |
| Land use change elasticity |  | 11.9% |  | 5.3% | |  | |
| Total CRP maintenance costs ($ billion) | 23.3 | 33.6 | 43.8% | 35.4 | | 5.3% | |

Notes: ^a^ This column shows the percentage changes in Scenario 2 relative to Scenario 1.

^b^ This column shows the percentage changes in Scenario 3 relative to Scenario 2.

**Table S4. Aggregated US Regions and Corresponding States.**

| **Region** | **States Included** |
| --- | --- |
| Northeast | Maine, Vermont, New Hampshire, Massachusetts, Connecticut, Rhode Island, New York, Pennsylvania, New Jersey, Delaware, Maryland, West Virginia |
|  |  |
| Midwest | Minnesota, Iowa, Missouri, Wisconsin, Illinois, Indiana, Michigan, Ohio |
|  |  |
| South | Arkansas, Kentucky, Tennessee, Virginia, Louisiana, Alabama, Mississippi, North Carolina, South Carolina, Georgia, Florida |
|  |  |
| Great Plains | South Dakota, North Dakota, Nebraska, Kansas, Oklahoma, Texas |
| West | Washington, Oregon, Nevada, Arizona, California, New Mexico, Montana, Idaho, Wyoming, Utah, Colorado |

**Table S5. Definition of Alternative Cases Considered for Sensitivity Analysis**

|  | **Cases** | **Definition** |
| --- | --- | --- |
| (1) | Higher price elasticity of soybean | The elasticity of soybean yield is 0.23 instead of 0 in the benchmark |
| (2) | Zero price elasticity of corn | The elasticity of corn yield is zero instead of 0.23 in the benchmark |
| (3) | 33% CRP productivity | CRP land productivity is uniformly 33% instead of the CRD-specific ratio of rental payment for a CRP acre to the average dryland cash rents in each CRD in the benchmark |
| (4) | 100% CRP productivity | CRP land productivity is uniformly 100% instead of the CRD-specific ratio of rental payment for a CRP acre to the average dryland cash rents in each CRD in the benchmark |
| (5) | Lower trend of yield | Trend rate of crop yields is 50% lower than the benchmark rate of 2.1 bu/yr for corn, 0.2 bu/yr for soybean, and 0.6 bu/yr for wheat |
| (6) | Higher trend of yield | Trend rate of crop yields is 50% higher than the benchmark rate of 2.1 bu/yr for corn, 0.2 bu/yr for soybean, and 0.6 bu/yr for wheat |
| (7) | Higher CRP soil rental rate | The returns of CRP acres reenrollment are 10% higher than the benchmark value |
| (8) | Lower CRP soil rental rate | The returns of CRP acres reenrollment are 10% lower than the benchmark value |
| (9) | Higher cost of cropland pasture | The cost of converting cropland pasture to cropland is 10% higher than the benchmark value |
| (10) | Lower cost of cropland pasture | The cost of converting cropland pasture to cropland is 10% lower than the benchmark value |
| (11) | Higher conversion efficiency | 1.8% annual growth rate of conversion efficiency of biofuel instead of fixed conversion efficiency of corn ethanol and biodiesel in the benchmark |
| (12) | Higher cost of pastureland | The cost of converting permanent pastureland to cropland is 10% higher than the benchmark value |
| (13) | Lower cost of pastureland | The cost of converting permanent pastureland to cropland is 10% lower than the benchmark value |

**
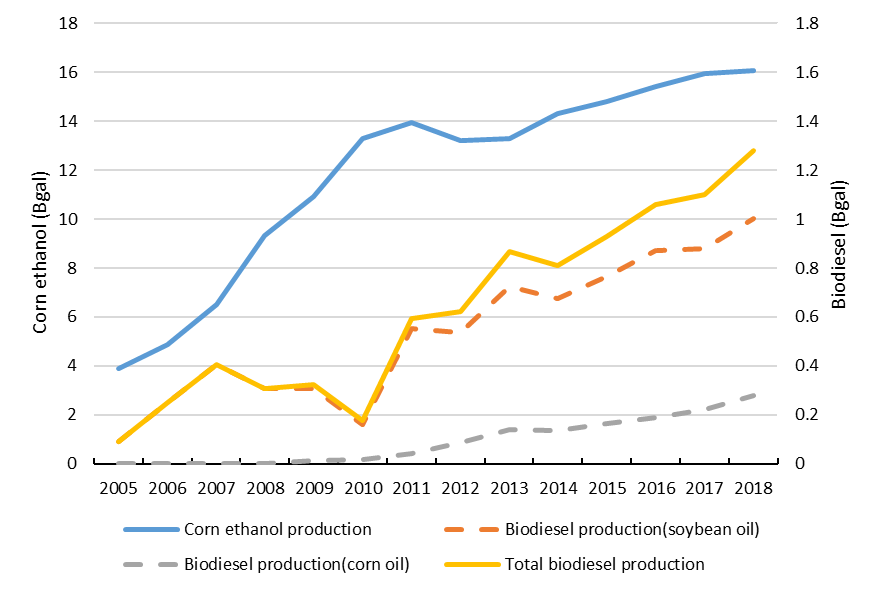
Figure S1. Corn Ethanol and Biodiesel Production from 2005 to 2018 (Source: EPA).** Blue line denotes total corn ethanol production (left axis); yellow line denotes total biodiesel production (right axis); orange and grey line represents soy biodiesel and corn oil biodiesel, respectively (right axis).


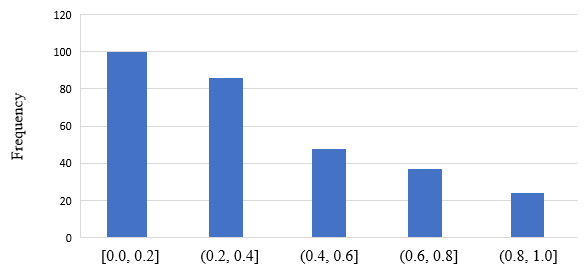


**Figure S2. Distribution of Ratio of Productivity of CRP Acres to Crop Acres Across CRDs**

**Figure S3. Model validation for scenario with no pastureland: Comparison of observed and simulated CRP and cropland acres.** (a) CRP land acres; (b) Total cropland acres. Red solid line denotes observed level of acres in each year, while blue solid line denotes simulated acreage under Scenario 3 **(**EtOH&BD Observed). Grey and yellow dashed line represents simulated acreage under Scenario 1 (EtOH&BD 2005) and Scenario 2 (EtOH Observed&BD 2005), respectively. Blue bars denote the percentage difference between simulated and observed acres. Green bars denote the percentage difference between acres in Scenario 1 and Scenario 2 ((Scenario 2 – Scenario 1)/Scenario1). Yellow bars denote the percentage difference in acres between Scenario 2 and Scenario 3((Scenario 3 – Scenario 2)/Scenario2).

**Figure S4. Model validation for scenario with additional pastureland** (a) CRP land acres; (b) Total cropland acres. Red solid line denotes observed level of acres in each year, while blue solid line denotes simulated acreage under Scenario 3 **(**EtOH&BD Observed). Grey and yellow dashed line represents simulated acreage under Scenario 1 (EtOH&BD 2005) and Scenario 2 (EtOH Observed&BD 2005), respectively. Blue bars denote the percentage difference between simulated and observed acres. Green bars denote the percentage difference between acres in Scenario 1 and Scenario 2 ((Scenario 2 – Scenario 1)/Scenario1). Yellow bars denote the percentage difference in acres between Scenario 2 and Scenario 3((Scenario 3 – Scenario 2)/Scenario2).

**Figure S5. Comparison of trends in simulated and observed key outcomes for scenario without pastureland.** Orange and blue line represents simulated and observed values, respectively. Grey bars denote the percentage difference between simulated and observed values.

**Figure S6. Comparison of trends in simulated and observed key outcomes for scenario with additional pastureland.** Orange and blue line represents simulated and observed values, respectively. Grey bars denote the percentage difference between simulated and observed values.

**Figure S7. Regional distribution of land use changes to corn (a), soybean (b), wheat (c) and alfalfa (d) due to corn ethanol production.** Dark blue bars denote the differences in amount of regular cropland used for crop production between Scenario 2 (EtOH Observed &BD 2005) and Scenario 1 (EtOH &BD 2005); Light blue bars denote the corresponding differences in noncropland. Positive values indicate an increase in land due to corn ethanol, while negative values indicate a reduction in land due to corn ethanol.

**Figure S8.** **Regional distribution of land use changes to corn (a), soybean (b), wheat (c) and alfalfa (d) due to additional biodiesel production.** Dark blue bars denote the differences in amount of regular cropland used for crop production between Scenario 3 (EtOH & BD Observed) and Scenario 2 (EtOH Observed & BD 2005); Light blue bars denote the corresponding differences in noncropland. Positive values indicate an increase in land due to biodiesel, while negative values indicate a reduction in land due to biodiesel.

**Figure S9.** **Sensitivity of model output to various modeling assumptions.** (a) Deviation in the percentage change due to corn ethanol under the benchmark parameters and under each alternative parametric assumption; (b) Deviation in the percentage change due to additional biodiesel under the benchmark parameters and under each alternative parametric assumption. A value close to zero indicates that model outcomes under the alternative parametric assumptions were close to those under the benchmark assumption.

1. **Model validation**

We validate the model by comparing the model simulated acres in CRP and in crop production in Scenario 3 (EtOH & BD Observed), with the observed levels of ethanol and biodiesel production as constraints over the 2007-2018 period. Figure S3 shows the results under the assumption that only cropland pasture and expiring CRP acres can convert to crop production. The corresponding results in the case where permanent pastureland can also convert to crop production are shown in Figure S4. Figure S3(a) shows the simulated and observed CRP acreage and the annual percentage deviations between the two (on the right-hand side axis). The deviation between simulated and observed CRP acres, during the 2007-2018 period, as a percentage of observed acres ranged between -4.8% and +1.5%. The total simulated decline in CRP acres in Scenario 3 between 2007 and 2018 was 13.6 million acres, which was also close (3.8% lower relative) to the observed decline in CRP acres during this period (14.1 million acres). We also estimate the simulated CRP acres under Scenario 1 (EtOH & BD 2005) and Scenario 2 (EtOH Observed & BD 2005). We find that even in the absence of expansion in biofuel production, CRP acres would have declined by 9.2 million acres over the 2007-2018 period, reflecting other policy changes such as those in acreage caps for the CRP and the land rental payments offered and market changes unrelated to biofuel policy. However, this decline was less than the decline of CRP acres in Scenarios with biofuel increase (EtOH Observed & BD 2005) and (EtOH & BD Observed), with the decline in the former Scenario being smaller than in latter Scenario. The inclusion of pastureland as potentially convertible to crop production, worsens the deviations between simulated CRP acres and observed CRP acres in the Scenario EtOH & BD Observed (Figure S4(a)) with deviations being greater than 10% in several years and overall reduction in CRP acres being 21.5% lower than observed.

The simulated outcomes are further validated by comparing the simulated total cropland acres in Scenario EtOH & BD Observed with observed total cropland acres (Figure S3(b)). The model simulations are a close fit to the observed data, with percentage deviations ranging between -1.2% and +4.3%. These deviations are very similar to those for the case with inclusion of pastureland conversion shown in Figure S4(b).

We also compare various model simulated outcomes over time with observed data in Figures S5 and S6. We find that the model predictions of acreages for corn, soybeans, and wheat, quantities of crop and fuel prices, production, and consumption are within ±10% (averagely 2%) and ±12% (averagely 3%) of observed data in most years with potential for cropland pasture only (Figure S5) and with potential for pastureland conversion (Figure S6), respectively.

1. **Sensitivity analysis**

We examined the sensitivity of our key results to alternative assumed values of several parameters in thirteen cases in the scenario without pastureland as available for conversion. In Case (1) and (2), we examined the robustness of our findings to variations in the price elasticities of corn yields. Miao *et al* (2016) provide empirical evidence that corn yields are price-elastic, but the effect of soybean price on soybean yields is not statistically significant. Our benchmark analysis assumed a price elasticity of corn yield of 0.23 following estimate from Miao *et al* (2016). The price elasticity of soybean yield is assumed as zero. We examined the extent to which alternative assumptions about this elasticity affects the impact of biofuel production on land use and crop prices. Specifically, in Case (1), we assumed the elasticity of soybean yields to be 0.23, the same as the value of corn yield. In Case (2), we assumed that the elasticity of corn yields to be zero. We then examined the sensitivity of model outcomes to the assumption of the ratio of the productivity of CRP acres being uniformly 33% and 100% in Cases (3) and (4), respectively. We also examined the sensitivity of our results to alternative assumptions about the trend rates of growth of crop yields. In the benchmark, we assume that the rates of growth of yields for corn, soybean, and wheat are 2.1, 0.2 and 0.6 bushels per year, respectively. In Cases (5) and (6), we assumed that these trend rates of crop yields were 50% lower and higher, respectively, than that in our benchmark case. We then examined the sensitivity of our results to alternative assumptions about the returns that CRP land would earn if they were converted to crop production in Cases (7)-(8). Specifically, we assumed that the returns that expiring CRP acres could earn upon reenrollment would be 10% higher and lower, respectively, than the payments assumed in the benchmark scenario, which was based on the observed soil rental payments being offered during the 2007-2018 period. In Case (9)-(10), we considered the possibility that 10% higher and lower conversion cost of cropland pasture. Furthermore, in case (11), we considered the improved conversion efficiency of biofuel production by assuming the annual improvement rate is 1.8% for both corn ethanol and biodiesel. This trend was determined based on the estimate by Irwin (2022). Lastly, in Case (12)-(13), we considered the possibility that 10% higher and lower conversion cost of permanent pastureland. We selected these scenarios to examine the robustness of our findings to uncertainties about key parameters.

We examined the effects of varying the assumptions described above on several key variables including total US cropland and corn and soybean price in 2018, the cumulative reduction in noncropland over the 2007-2018 period, the discounted value of the total CRP maintenance cost over the 2007-2018 period and the land use change intensity (i.e., million acres per billion gallons). For each of these variables, we first computed the percentage changes in value under Scenario 2 (EtOH Observed & BD 2005) relative to Scenario 1(EtOH & BD 2005) and under Scenario 3 (EtOH & BD Observed) relative to Scenario 2 (EtOH Observed & BD 2005). We then computed the differences in these percentage changes for the five outcome variables under each of these alternative cases relative to those obtained under the benchmark case. Figure S9(a) shows the deviations in estimated percentage change in total US cropland due to the increase in corn ethanol production.

We find that the effect of alternative assumptions on the change in cropland in Scenario 2 (EtOH Observed & BD 2005) relative to Scenario 1 (EtOH & BD 2005) is between -0.2% and 0.3% and most sensitive to assumptions about yield growth rates. The effect of alternative parameters on noncropland is between ±2% while that on corn price is between ±3% and on soybean price is between -3% and 2%. The largest variation across the alternative cases is found in the CRP maintenance costs which ranges between ±6.5% and are most sensitive to assumptions about productivity of CRP land and CRP rental rates. Uniform 33% CRP land productivity leads to a 6.4% increase of CRP maintenance costs, while 10% lower rental payments decrease CRP maintenance costs by 6.7% relative to the benchmark case. Estimates of land use intensity (million acres per billion gallons) are within +4% and (-6%) of the estimates in the benchmark case. Growth rate of crop yields and land conversion cost of cropland pasture are two most sensitive parameters. Overall, results, particularly those on the extent of land use change that can be attributed to corn ethanol are robust to the various parametric assumptions considered here.

Figure S9(b) shows the deviations in estimated percentage increases in total US cropland due to the additional biodiesel production across the thirteen alternative cases. We find that the effect of alternative assumptions on the change in cropland in Scenario 3 (EtOH & BD Observed) relative to Scenario 2 (EtOH Observed & BD 2005) is less than 0.1%. The effect on noncropland is within ±0.3% across the various alternative cases considered here. The largest magnitude of change due to changes in the assumptions considered here is in the price of corn and soybeans; the effect on corn prices ranges between -2% and 5% and on soybean prices between -2.5% and 4.4%. CRP maintenance costs are most sensitive to assumption of CRP rental payments. 10% increase of CRP rental payments will lead to 1.7% higher CRP maintenance costs and 10% lower CRP rental payments will decrease CRP maintenance costs by 1.5%. Land use intensity of biodiesel could be about 9% higher with a halving of the growth rate of yields and 9% lower with a 10% increase in cost of conversion of cropland pasture.

**References**

Antares Group Inc 2009 National biorefinery siting model draft final report-task 2: technologies for biofuels production

Argonne National Laboratory 2020 GREET model https://greet.es.anl.gov (accessed Aug 1, 2021)

Beach R H and McCarl B A 2010 *Agricultural and forestry impacts of the energy independence and security act: FASOM results and model description* (Final report prepared for US Environmental Protection Agency, Washington DC)

Chen L, Blanc-Betes E, Hudiburg T W, Hellerstein D, Wallander S, DeLucia E H and Khanna M 2021 Assessing the returns to land and greenhouse gas savings from producing energy crops on Conservation Reserve Program land *Environ. Sci. Technol.* **55** 1301–9

Chen X, Huang H, Khanna M and Önal H 2014 Alternative transportation fuel standards: Welfare effects and climate benefits *Journal of Environmental Economics and Management* **67** 241–57

Chen X and Khanna M 2018 Effect of corn ethanol production on Conservation Reserve Program acres in the US *Applied Energy* **225** 124–34

EPA 2010 Renewable Fuel Standard Program (RFS2) Regulatory Impact Analysis, EPA-420-R-10-00, Available on line at http://www.epa.gov/otaq/fuels/renewablefuels/index.htm, US Environmental Protection Agency, Washington, DC.

Irwin S 2022 Trends in the operational efficiency of the U.S. ethanol industry: 2021 Update. Farmdoc daily (12): 14, Department of Agricultural and Consumer Economics, University of Illinois at Urbana-Champaign

Jiang C, Guan K, Khanna M, Chen L and Peng J 2021 Assessing marginal land availability based on land use change information in the contiguous United States *Environmental Science & Technology* Online: https://pubs.acs.org/doi/abs/10.1021/acs.est.1c02236

Miao R, Khanna M and Huang H 2016 Responsiveness of Crop Yield and Acreage to Prices and Climate *American Journal of Agricultural Economics* **98** 191–211

Mueller S 2010 2008 National dry mill corn ethanol survey *Biotechnol Lett* **32** 1261–4

Office of the Federal Register 2018 Federal Register Vol. 83 No. 149. https://thefederalregister.org/83-FR/Issue-149. (Accessed on Sep 3, 2022)

Stubbs M 2014 Conservation Reserve Program (CRP): Status and Issues. Congressional Research Services Report R42783.

USDA 2020 Statistics by subject-Animal & products. Available online at: https://www.nass.usda.gov/Statistics_by_Subject/index.php?sector=CROPS

USDA/NASS 2014 2012 Census of Agriculture, United States Summary and State Data. Report AC-12-A-51. https://www.nass.usda.gov/Publications/AgCensus/2012/ (accessed Aug 1, 2021)
